# 

**Published:** 1909-02

**Authors:** 


### CONTENTS.

ORIGINAL COMMUNICATIONS—

Inaugural Address Before the Fulton County Medical Society. By Cyrus W. Strickler, M. D., Atlanta, Ga. .. .. .. .. .. .. .. .. 555

Remarks on the President's Address. By. Dr. John C. Olmsted, Atlanta, Ga. .. .. .. .. .. 558

Blood in the Urine—Some Cases. By Alfred L. Fowler, M. D., Atlanta, Ga. .. .. .. .. .. 562

Acute Traumatic Tetanus Treated by Magnesium Sulphate. By Aime Paul Heineck, Chicago, Ill. 569

"Tricks in all Trades but Ourn." By Arthur G. Hobs, M. D., Atlanta, Ga. .. .. .. .. .. 580

Comments on two Recent Cases of Gastro-Enterostomy. B. Edward G. Jones, M. D., Atlanta, Ga. 582

Aesthetic Alimination. By Geo. M. Niles, M. D., Atlanta, Ga. .. .. .. .. .. .. .. 589

EDITORIALS .. .. .. .. .. .. .. .. .. .. .. .. 592

NEWS AND NOTES .. .. .. .. .. .. .. .. .. .. .. 598

BOOK REVIEWS .. .. .. .. .. .. .. .. .. .. .. 607

